# Emerging trends in geospatial artificial intelligence (geoAI): potential applications for environmental epidemiology

**DOI:** 10.1186/s12940-018-0386-x

**Published:** 2018-04-17

**Authors:** Trang VoPham, Jaime E. Hart, Francine Laden, Yao-Yi Chiang

**Affiliations:** 1 Department of Epidemiology, Harvard T.H. Chan School of Public Health, 677 Huntington Avenue, Boston, MA 02115 USA; 2 Channing Division of Network Medicine, Department of Medicine, Brigham and Women’s Hospital and Harvard Medical School, 181 Longwood Avenue, Boston, MA 02115 USA; 3 Exposure, Epidemiology and Risk Program, Department of Environmental Health, Harvard T.H. Chan School of Public Health, 677 Huntington Avenue, Boston, MA 02115 USA; 4 Spatial Sciences Institute, University of Southern California, 3616 Trousdale Parkway AHF B55, Los Angeles, CA 90089 USA

**Keywords:** Geospatial artificial intelligence, geoAI, Spatial data science, Machine learning, Deep learning, Data mining, Remote sensing, Environmental epidemiology, Exposure modeling

## Abstract

Geospatial artificial intelligence (geoAI) is an emerging scientific discipline that combines innovations in spatial science, artificial intelligence methods in machine learning (e.g., deep learning), data mining, and high-performance computing to extract knowledge from spatial big data. In environmental epidemiology, exposure modeling is a commonly used approach to conduct exposure assessment to determine the distribution of exposures in study populations. geoAI technologies provide important advantages for exposure modeling in environmental epidemiology, including the ability to incorporate large amounts of big spatial and temporal data in a variety of formats; computational efficiency; flexibility in algorithms and workflows to accommodate relevant characteristics of spatial (environmental) processes including spatial nonstationarity; and scalability to model other environmental exposures across different geographic areas. The objectives of this commentary are to provide an overview of key concepts surrounding the evolving and interdisciplinary field of geoAI including spatial data science, machine learning, deep learning, and data mining; recent geoAI applications in research; and potential future directions for geoAI in environmental epidemiology.

## Background

Spatial science, also referred to as geographic information science, plays an important role in many scientific disciplines as it seeks to understand, analyze, and visualize real-world phenomena according to their locations. Spatial scientists apply technologies such as geographic information systems (GIS) and remote sensing to spatial (e.g., georeferenced) data to achieve these objectives – to identify and make sense of patterns in space. Tied to the current era of big data is the real-time generation of spatial big data, which have become ubiquitously available from geotagged social media posts on Twitter to environmental sensors collecting meteorological information [1]. It has been suggested that at least 80% of all data are geographic in nature, as the majority of information around us can be georeferenced [1]. By this measure, 80% of the 2.5 exabytes (2,500,000,000 gigabytes) of big data generated everyday is geographic [2]. Data science, and by extension spatial data science, are still evolving fields that provide methods to organize how we think about and approach generating new knowledge from (spatial) big data.

The scientific field of geospatial artificial intelligence (geoAI) was recently formed from combining innovations in spatial science with the rapid growth of methods in artificial intelligence (AI), particularly machine learning (e.g., deep learning), data mining, and high-performance computing to glean meaningful information from spatial big data. geoAI is highly interdisciplinary, bridging many scientific fields including computer science, engineering, statistics, and spatial science. The innovation of geoAI partly lies in its applications to address real-world problems. In particular, geoAI applications were showcased at the inaugural 2017 Association of Computing Machinery (ACM) Special Interest Group on Spatial Information (SIGSPATIAL) International Workshop on GeoAI: AI and Deep Learning for Geographic Knowledge Discovery (the steering committee was led by the U.S. Department of Energy Oak Ridge National Laboratory Urban Dynamics Institute), which included advances in remote sensing image classification and predictive modeling for traffic. Further, the application of AI technologies for knowledge discovery from spatial data reflects a recent trend as demonstrated in other scientific communities including the International Symposium on Spatial and Temporal Databases. These novel geoAI methods can be used to address human health-related problems, for example, in environmental epidemiology [3]. In particular, geoAI technologies are beginning to be used in the field of environmental exposure modeling, which is commonly used to conduct exposure assessment in these studies [4]. Ultimately, one of the overarching goals for integrating geoAI with environmental epidemiology is to conduct more accurate and highly resolved modeling of environmental exposures (compared to conventional approaches), which in turn would lead to more accurate assessment of the environmental factors to which we are exposed, and thus improved understanding of the potential associations between environmental exposures and disease in epidemiologic studies. Further, geoAI provides methods to measure new exposures that have been previously difficult to capture.

The purpose of this commentary is to provide an overview of key concepts surrounding the emerging field of geoAI; recent advances in geoAI technologies and applications; and potential future directions for geoAI in environmental epidemiology.

## Distinguishing between the buzzwords: the spatial in big data and data science

Several key concepts are currently at the forefront of understanding the geospatial big data revolution. Big data, such as electronic health records and customer transactions, are generally characterized by a high volume of data; large variety of data sources, formats, and structures; and a high velocity of new data creation [5–7]. As a consequence, big data require specialized methods and techniques for processing and analysis. Data science broadly refers to methods to provide new knowledge from the rigorous analysis of big data, integrating methods and concepts from disciplines including computer science, engineering, and statistics [8, 9]. The data science workflow generally resembles an iterative process of data import and processing, followed by cleaning, transformation, visualization, modeling, and finally communication of results [10].

Spatial data science is a niche and still forming field focused on methods to process, manage, analyze, and visualize spatial big data, providing opportunities to derive dynamic insights from complex spatial phenomena [11]. Spatial data science workflows are comprised of steps for data manipulation, data integration, exploratory data analysis, visualization, and modeling – and are specifically applied to spatial data often using specialized software for spatial data formats [12]. For example, a spatial data science workflow may include data wrangling using open source solutions such as the Geospatial Data Abstraction Library (GDAL), scripting in R, Python, and Spatial SQL for spatial analyses facilitated by high-performance computing (e.g., querying big data stored on a distributed data infrastructure through cloud computing platforms such as Amazon Web Services for analysis; or spatial big data analytics conducted on a supercomputer), and geovisualization using D3. Spatial data synthesis is considered an important challenge in spatial data science, which includes issues related to spatial data aggregation (of different scales) and spatial data integration (harmonizing diverse spatial data types related to format, reference, unit, etc.) [11]. Advances in cyberGIS (defined as GIS based on advanced cyberinfrastructure and e-science) – and more broadly high-performance computing capabilities for high-dimensional data – have played an integral role in transforming our capacity to handle spatial big data and thus for spatial data science applications. For example, a National Science Foundation-supported cyberGIS supercomputer called ROGER was created in 2014, which enables the execution of geospatial applications requiring advanced cyberinfrastructure through high-performance computing (e.g., > 4 petabytes of high-speed persistent storage), graphics processing unit (GPU)-accelerated computing, big data-intensive subsystems using Hadoop and Spark, and Openstack cloud computing [11, 13].

As spatial data science continues to evolve as a discipline, spatial big data are constantly expanding, with two prominent examples being volunteered geographic information (VGI) and remote sensing. The term VGI encapsulates user-generated content with a locational component [14]. In the past decade, VGI has seen an explosion with the advent and continued expansion of social media and smart phones, where users can post and thus create geotagged tweets on Twitter, Instagram photos, Snapchat videos, and Yelp reviews [15]. Usage of VGI should be accompanied by an awareness of potential legal issues including but not limited to intellectual property, liability, and privacy for the operator, contributor, and user of VGI [16]. Remote sensing is another type of spatial big data capturing characteristics of objects from a distance such as imagery from satellite sensors [17]. Depending on the sensor, remote sensing spatial big data can be expansive in both its geographic coverage (spanning the entire globe) as well as its temporal coverage (with frequent revisit times). In recent years, we have seen an enormous increase in satellite remote sensing big data as private companies and governments continue to launch higher resolution satellites. For example, DigitalGlobe collects over 1 billion km^2^ of high-resolution imagery each year as part of its constellation of commercial satellites including the WorldView and GeoEye spacecraft [18]. The U.S. Geological Survey and NASA Landsat program has continually launched earth-observing satellites since 1972, with spatial resolutions as fine as 15 m and increasing spectral resolution with each subsequent Landsat mission (e.g., Landsat 8 Operational Land Imager and Thermal Infrared Sensor launched in 2013 are comprised of 9 spectral bands and 2 thermal bands) [19].

## Geospatial artificial intelligence (geoAI): nascent origins

Data science involves the application of methods in scientific fields such as artificial intelligence (AI) and data mining. AI refers to machines that make sense of the world, automating processes that create scalable insights from big data [5, 20]. Machine learning is a subset of AI that focuses on computers acquiring knowledge to iteratively extract information and learn from patterns in raw data [20, 21]. Deep learning is a cutting-edge type of machine learning that draws inspiration from brain function, representing a flexible and powerful way to enable computers to learn from experience and understand the world as a nested hierarchy of concepts, where the computer is able to learn complicated concepts by building them from simpler concepts [20]. Deep learning has been applied to natural language processing, computer vision, and autonomous driving [20, 22]. Data mining refers to techniques to discover new and interesting patterns from large datasets such as identifying frequent itemsets in online transaction records [23]. Many techniques for data mining were developed as part of machine learning [24]. Applications of data mining techniques include recommender systems and cohort detection in social networks.

Geospatial artificial intelligence (geoAI) is an emerging science that utilizes advances in high-performance computing to apply technologies in AI, particularly machine learning (e.g., deep learning) and data mining to extract meaningful information from spatial big data. geoAI is both a specialized field within spatial science because particular spatial technologies, including GIS, must be used to process and analyze spatial data, and an applied type of spatial data science, as it is specifically focused on applying AI technologies to analyze spatial big data. The first-ever International Workshop on geoAI organized as part of the 2017 ACM SIGSPATIAL International Conference on Advances in Geographic Information Systems brought together scientists across diverse disciplines, including geoscientists, computer scientists, engineers, and entrepreneurs to discuss the latest trends in deep learning for geographical data mining and knowledge discovery. Featured geoAI applications included deep learning architectures and algorithms for feature recognition in historical maps [25]; multi-sensor remote sensing image resolution enhancement [26]; and identification of the semantic similarity in VGI attributes for OpenStreetMap [27]. The geoAI Workshop is one example of the recent trend in the application of AI to spatial data. For example, AI research has been presented at the International Symposium on Spatial and Temporal Databases, which features research in spatial, temporal, and spatiotemporal data management and related technologies.

## Opportunities for geoAI in environmental epidemiology

Given the advances and capabilities on display in recent research, we can begin to connect the dots regarding how geoAI technologies can be specifically applied to environmental epidemiology. To determine the factors to which we may be exposed and thus may influence health, environmental epidemiologists implement direct methods of exposure assessment, such as biomonitoring (e.g., measured in urine), and indirect methods, such as exposure modeling. Exposure modeling involves the development of a model to represent a particular environmental variable using various data inputs (such as environmental measurements) and statistical methods (such as land use regression and generalized additive mixed models) [28]. Exposure modeling is a cost-effective approach to assess the distribution of exposures in particularly large study populations compared to applying direct methods [28]. Exposure models include basic proximity-based measures (e.g., buffers and measured distance) to more advanced modeling such as kriging [3]. Spatial science has been critical in exposure modeling for epidemiologic studies over the past two decades, enabling environmental epidemiologists to use GIS technologies to create and link exposure models to health outcome data using geographic variables (e.g., geocoded addresses) to investigate the effects of factors such as air pollution on the risk of developing diseases such as cardiovascular disease [29, 30].

geoAI methods and big data infrastructures (e.g., Spark and Hadoop) can be applied to address challenges surrounding exposure modeling in environmental epidemiology – including inefficiency in computational processing and time (particularly when big data are compounded with large geographic study areas) and data-related constraints that affect spatial and/or temporal resolution. For example, previous exposure modeling efforts have often been associated with coarse spatial resolutions, impacting the extent to which the exposure model is able accurately estimate individual-level exposure (i.e., exposure measurement error), as well as limitations in temporal resolution which may result in failure to capture exposures during time windows relevant to developing the disease of interest [28]. Advances in geoAI enable accurate, high-resolution exposure modeling for environmental epidemiologic studies, especially regarding high-performance computing to handle big data (big in space and time; spatiotemporal) as well as developing and applying machine and deep learning algorithms and big data infrastructures to extract the most meaningful and relevant pieces of input information to, for example, predict the amount of an environmental factor at a particular time and location.

A recent example of geoAI in action for environmental exposure assessment was a data-driven method developed to predict particulate matter air pollution < 2.5 μm in diameter (PM_2.5_) in Los Angeles, CA, USA [4]. This research utilized the Pediatric Research using the Integrated Sensor Monitoring Systems (PRISMS) Data and Software Coordination and Integration Center (DSCIC) infrastructure [4, 31]. A spatial data mining approach using machine learning and OpenStreetMap (OSM) spatial big data was developed to enable selection of the most important OSM geographic features (e.g., land use and roads) predicting PM_2.5_ concentrations. This spatial data mining approach addresses important issues in air pollution exposure modeling regarding the spatial and temporal variability of the relevant “neighborhood” within which to determine how and which factors influence predicted exposures (spatial nonstationarity is discussed later). Using millions of geographic features available from OSM, the algorithm to create the PM_2.5_ exposure model first identified U.S. Environmental Protection Agency (EPA) air monitoring stations that exhibited similar temporal patterns in PM_2.5_ concentrations. The algorithm next trained a random forest model (a popular machine learning method using decision trees for classification and regression modeling) to generate the relative importance of each OSM geographic feature. This was performed by determining the geo-context, or which OSM features and within what distances (e.g., 100 m vs. 1000 m radius buffers) are associated with air monitoring stations (and their measured PM_2.5_ levels) characterized by a similar temporal pattern. Finally, the algorithm trained a second random forest model using the geo-contexts and measured PM_2.5_ at the air monitoring stations to predict PM_2.5_ concentrations at unmeasured locations (i.e., interpolation). Prediction errors were minimized through incorporating temporality of measured PM_2.5_ concentrations in each stage of the algorithm, although modeling would have been improved with time-varying information on predictors. The model predictive performance using measured PM_2.5_ levels at the EPA air monitoring stations as the gold standard showed an improvement compared to using inverse distance weighting, a commonly used spatial interpolation method [4]. Through this innovative approach, Lin et al. (2017) developed a flexible spatial data mining-based algorithm that removes the need for a priori selection of predictors for exposure modeling, as important predictors may depend on the specific study area and time of day – essentially letting the data decide what is important for exposure modeling [4].

## Future directions

The application of geoAI, specifically using machine learning and data mining, to air pollution exposure modeling described in Lin et al. (2017) demonstrates several key advantages for exposure assessment in environmental epidemiology [4]. geoAI algorithms can incorporate large amounts of spatiotemporal big data, which can improve both the spatial and temporal resolutions of the output predictions, depending on the spatial and temporal resolutions of the input data and/or downscaling methodologies to create finer resolution data from relatively coarser data. Beyond incorporating high-resolution big data that are being generated in real-time, existing historical big data, such as Landsat satellite remote sensing imagery from 1972 to present, can be used within geoAI frameworks for historical exposure modeling – advantageous to studying chronic diseases with long latency periods. This seamless usage and integration of spatial big data is facilitated by high-performance computing capabilities, which provide a computationally efficient approach to exposure modeling using high-dimensional data compared to other existing time-intensive approaches (e.g., dispersion modeling for air pollution) that may lack such computational infrastructures.

Further, the flexibility of geoAI workflows and algorithms can address properties of environmental exposures (as spatial processes) that are often ignored during modeling such as spatial nonstationarity and anisotropy [32]. Spatial nonstationarity occurs when a global model is unsuitable for explaining a spatial process due to local variations in, for example, the associations between the spatial process and its predictors (i.e., drifts over space) [32, 33]. Lin et al. (2017) addressed spatial nonstationarity through creating unique geo-contexts using the OSM geographic features for air monitoring stations grouped into similar temporal patterns. Anisotropic spatial processes are characterized by directional effects [32], for example, the concentration of an air pollutant may be affected by wind speed and wind direction [34]. The flexibility in geoAI workflows naturally allows for scalability to use and modify algorithms to accommodate more big data (e.g., unconventional datasets such as satellite remote sensing to derive city landscapes for air quality dispersion modeling), different types of big data, and extending modeling to predict different environmental exposures in different geographic areas. An additional facet of this flexibility includes the ability for many machine learning and data mining techniques to be conducted without a high degree of feature engineering, enabling the inclusion of large amounts of big data, for example greater amounts of surrogate variables when direct measures are unavailable. In addition, another potential area of application for geoAI involves algorithm development to quickly and accurately classify and identify objects from remote sensing data that have been previously difficult to capture, for example, features of the built environment based on spectral and other characteristics to generate detailed 3D representations of city landscapes.

Ultimately, geoAI applications for environmental epidemiology move us closer to achieving the goal of providing a highly resolved and more accurate picture of the environmental exposures to which we are exposed, which can be combined with other relevant information regarding health outcomes, confounders, etc., to investigate whether a particular environmental exposure is associated with a particular outcome of interest in an epidemiologic study. However, as with any exposure modeling endeavor, there must be careful scrutiny of data quality and consideration of data costs. In the context of the Lin et al. (2017) study [4], although this type of data-driven approach enables flexibility in the amount of spatial big data that can be incorporated and in allowing the data to determine model inputs, it is incumbent on the spatial data scientist to evaluate data quality and assess whether or not the spatial resolution and other data attributes are useful for the application at hand – to avoid what is referred to as garbage in, garbage out (GIGO) in computer science. Related to data quality is the importance of data-driven approaches to be balanced against the need for domain-specific expertise. For example, if a particular variable that is a known predictor of PM_2.5_ (irrespective of time and space) is not selected as part of a data-driven method for inclusion into exposure modeling, this may require modifications to the algorithm, evaluation of the input data, etc. Finally, as a currently evolving field, geoAI requires the expertise of multiple disciplines, including epidemiology, computer science, engineering, and statistics, to establish best practices for how to approach environmental exposure modeling given the complexities introduced by the biological, chemical, and physical properties of different environmental exposures, wide-ranging algorithms that can be developed and applied, and heterogeneous spatial big data characterized by varying scales, formats, and quality.

## Conclusions

geoAI is an emerging interdisciplinary scientific field that harnesses the innovations of spatial science, artificial intelligence (particularly machine learning and deep learning), data mining, and high-performance computing for knowledge discovery from spatial big data. geoAI traces part of its roots from spatial data science, which is an evolving field that aims to help organize how we think about and approach processing and analyzing spatial big data. Recent research demonstrates movement towards practical applications of geoAI to address real-world problems from feature recognition to image enhancement. geoAI offers several advantages for environmental epidemiology, particularly for exposure modeling as part of exposure assessment, including the capability to incorporate large amounts of spatial big data of high spatial and/or temporal resolution; computational efficiency regarding time and resources; flexibility in accommodating important features of spatial (environmental) processes such as spatial nonstationarity; and scalability to model different environmental exposures in different geographic areas. Potential future geoAI applications for environmental epidemiology should utilize cross-disciplinary approaches to developing and establishing rigorous and best practices for exposure modeling that includes careful consideration of data quality and domain-specific expertise.

## Abbreviations

ACM: Association of Computing Machinery
AI: artificial intelligence
DSCIC: Data and Software Coordination and Integration Center
EPA: Environmental Protection Agency
geoAI: geospatial artificial intelligence
GIGO: garbage in, garbage out
GIS: geographic information system
GPU: graphics processing unit
OSM: OpenStreetMap
PM_2.5_: particulate matter air pollution < 2.5 μm in diameter
PRISMS: Pediatric Research using the Integrated Sensor Monitoring Systems
SIGSPATIAL: Special Interest Group on Spatial Information
VGI: volunteered geographic information
